# Phytochemical profiling and allelopathic effect of garlic essential oil on barnyard grass (*Echinochloa crusgalli* L.)

**DOI:** 10.1371/journal.pone.0272842

**Published:** 2023-04-25

**Authors:** Haodong Bai, Xianzhi Ni, Jincai Han, Dingfeng Luo, Yihong Hu, Chenzhong Jin, Zuren Li

**Affiliations:** 1 Collaborative Innovation Center for Field Weeds Control, Hunan University of Humanities and Science, Loudi, Hunan, China; 2 Hunan Provincial Key Laboratory for Biology and Control of Weeds, Hunan Academy of Agricultural Sciences, Changsha, Hunan, China; Bangabandhu Sheikh Mujibur Rahman Agricultural University, BANGLADESH

## Abstract

In agriculture, barnyard grass (*Echinochloa crusgalli* L.) is one of the most harmful weeds in rice fields now. In order to identify active ingredients which had inhibiting effect on barnyard grass (Echinochloa crusgalli L.), we evaluated several possible natural plant essential oils. Essential oils from twelve plant species showed inhibitory activity against barnyard grass seedlings and root length. The garlic essential oil (GEO) had the most significant allelopathic effect (EC_50_ = 0.0126 g mL^-1^). Additionally, the enzyme activities of catalase (CAT), peroxidase (POD) and superoxide dismutase (SOD) increased during the first 8 hours of treatment at a concentration of 0.1 g mL^-1^ and then declined. The activities of CAT, SOD and POD increased by 121%, 137% and 110% (0–8h, compared to control), and decreased (8–72h, compared to the maximum value) by 100%, 185% and 183%, respectively. The total chlorophyll content of barnyard grass seedlings decreased by 51% (0–72h) continuously with the same dosage treatment. Twenty constituents of GEO were identified by gas chromatography-mass spectrometry, and the herbicidal activity of two main components (diallyl sulfide and diallyl disulfide) was evaluated. Results showed that both components had herbicidal activity against barnyard grass. GEO had a strong inhibitory effect (~88.34% inhibition) on barnyard grass growth, but safety studies on rice showed it did not have much inhibitory effect on rice seed germination. Allelopathy of GEO provide ideas for the development of new plant-derived herbicides.

## Introduction

The barnyard grass, *Echinochloa crusgalli* L. (Family: Poaceae) is one of the most difficult malignant weeds to control in paddy fields because its biological characteristics are very similar to rice and also mimics the characteristics of crops [1]. Meanwhile barnyard grass and rice are great competitors in the fields [2]. At present, there are no highly selective and safe bio-herbicides for controlling barnyard grass in rice fields. Evaluation of allelochemicals, especially their inhibition functions, is an effective method for identifying potential herbicides [3]. Plant essential oils can function as allelochemicals and therefore serve as a resource pool which can provide effective allelochemicals [4]. For example, the application of 200 μL L^-1^ of *Cleome droserifolia* (Forssk.) delile. EO (sesquiterpene was a major class and represented by 61.97% of the total essential oil) to *Trifolium repen* reduced its root length by 42.9% compared to the control [5]. At present, there are very few essential oils used for barnyard grass control [6, 7]. Thus, it is pertinent to screen for additional essential oils for the efficient control of barnyard grass.

In this study, the essential oils of these twelve plant species were utilized to treat barnyard grass to assay their herbicidal effects. Many studies have shown that Liliaceae plants contain polysaccharides, flavonoids, polyphenols, saponins, amino acids, alkaloids, thioethers, steroids and other components [8]. Past research on the activity of these active ingredients has mainly been on their anti-tumor, anti-inflammatory, bacteriostatic activities [9]. Other active functions of Liliaceae essential oils have not been studied so far, and reports on its application in agricultural weed control is almost absent in the literature. In this study, the herbicidal activities of the major compositions of GEO and their herbicidal activities were determined using gas chromatography-mass spectrometry (GC-MS). This study provides insight into the effects of GEO on the antioxidant enzymes activities and chlorophyll content in barnyard grass seedlings, as well its safety on rice.

## Materials and methods

### Material and culture conditions

The seeds of barnyard grass (*Echinochloa crusgalli* L.) and rice (Long Liang You534) used in the experiment were collected from a farm at Gaoqiao Base in Changsha city of Hunan Province, China (113.03 E, 28.18 N). Twenty seeds from each species were placed into a plastic cup containing 15 g soil. These plants were grown in an artificial greenhouse under a 12 h photoperiod (light / dark cycle), 150 μmol m^-2^ s^-1^ (light intensity) and 28 / 23°C (day / night temperature regime). The faster growth of barnyard grass compared to rice in the field, uniformly-grown barnyard grass seedlings at the 3-leaves stage and rice seedlings at the first and second leaf stage were selected for subsequent use in the herbicide-activity tests. All the bioassays and enzyme activity tests were repeated three times.

### Tested plant essential oils

Twelve kinds of plant essential oils were purchased from Jiangxi Yisengyuan Plant Fragrance Co., Ltd. Including: garlic essential oil-GEO # 9 (*Allium sativum*, Liliaceae), tangerine peel essential oil-TEO # 14 (*Citrus reticulatae*, Rutaceae), spearmint essential oil-SEO # 15 (*Mentra spicata*, Labiaceae), yellow wormwood leaf essential oil-YEO # 16 (*Artemisia argyi*, Compositae), *Litsea cubeba* essential oil-LCEO # 17 (Compositae), pine needle essential oil-PEO # 18 (*Pinus sylvestris*, Pinaceae), cedar essential oil-CEO # 19 (*Cedrus atlantica*, Pinaceae), lemon grass essential oil-LGEO # 20 (*Cymbopogan flexuosus*, Poaceae), rosemary essential oil-REO # 21 (*Rosmarinus officinalis*, Lamiaceae), juniper essential oil-JEO # 22 (*Juniperus communis*, Cupressaceae), geranium essential oil-GEEO # 23 (*Pelargonium graveolens*, Geraspinaceae), star anise essential oil-SAEO # 24 (*Illicium verum*, Illiciaceae).

### Bioassay of essential oil effects on barnyard grass

The herbicidal activity of plant derived essential oil solutions (essential oil and 2.5% Tween 80 and 2.5% dimethylformamide, distilled water added to 10 mL) was evaluated by spraying on barnyard grass. The concentrations of the twelve plant essential oils sprayed on the 3-leaf stage barnyard grass seedlings were 0, 0.01, 0.03, 0.05, 0.08 and 0.1 g mL^-1^ in the preliminary screening. The concentrations of the GEO sprayed on the 3-leaf stage barnyard grass seedlings were 0, 0.001, 0.005, 0.01, 0.03, and 0.1 g mL^-1^. A completely randomized design with three replications was applied to all of the experiments. Three untreated pots served as control and the seedlings weights were recorded after seven days. Seven days after spraying, the treated plants were examined for injury [7]. The seedlings fresh weight inhibition ratio was calculated according to the formula: [1-(experiment group/control group)×100%] [10].

To evaluate effect of twelve plant derived essential oil on the growth of barnyard grass root length. Fifteen healthy full *E*. *crus-galli* seed were germinated in Petri dishes (diameter = 9 cm) with lined with a single layer of Whatman # 2 filter paper and grown in the greenhouse (12 h photoperiod (light / dark cycle), 150 μmol m^-2^ s^-1^ (light intensity) and 28 / 23°C (day / night temperature regime)). Twelve plant derived essential oil (essential oil and 2.5% Tween 80 and 2.5% dimethylformamide, final concentration were 0.1g L^-1^) were added to Petri dish, to which 1mL of water was poured every day until the seventh day. A completely randomized design with three replications was applied to all of the experiments. The length of roots were measured in the various treatments, and the inhibition rate were calculated after 7 days of treatment as follows: Inhibition rate (%) = [100% × (length of roots of control-length of roots of treatment)/length of roots of control] [11].

### Evaluation of the safety of GEO on rice

To evaluate that GEO was safe on rice, a seed germination experiment was carried out. In order to ensure 100% germination, the seeds were accelerating germination in the germination chamber and then transferred to greenhous. For this, 15 selected rice seeds were placed in a Petri dish and placed under a constant temperature of 35°C and light in an incubator for 1 day. The Petri dishes were moved to an artificial greenhouse under a 12 h photoperiod (light / dark cycle), 150 μmol m^-2^ s^-1^ (light intensity) and 28 / 23°C (day/night temperature regime). Different concentrations of GEO (0.01, 0.03, 0.05, 0.1 g mL^-1^) were added to each Petri dish containing the 15 rice seeds, to which 1mL of water was poured every day until the seventh day. Rice seeds in the control group were cultivated normally and their germination conditions carefully observed. A completely randomized design with three replications was applied to all of the experiments.

In the seedling tests, the safety of the plant-derived oil solutions (essential oil and 2.5% Tween 80 and 2.5% dimethylformamide, distilled water added to 10 mL) was evaluated by spraying on rice at the first and second leaf stage. The concentrations of the GEO sprayed were 0.01, 0.03, 0.05, 0.1 g mL^-1^. The treated rice seedlings were kept in an artificial greenhouse under the conditions described above. Three untreated pots served as the control and the seedlings weights were recorded after seven days. The treated plants were examined for injury, 7 days after spraying [7]. A completely randomized design with three replications was applied to all of the experiments. Parameters were calculated as follows: germination percentage = (number of germinated seeds/total number of seeds)×100 [12]. The seedlings fresh weight inhibition ratio was calculated according to the formula: [1-(experiment group/control group)×100%] [10].

### Gas chromatography-mass spectrometry (GC-MS) analysis

For all analysis, the main instrument used was the Shimazu QP5050A GC-MS, equipped with the Shimazu AOC-20i automatic sampler. Retention indices values were calculated according to the equation described by Mazur [13].

The chromatographic conditions for the GEO were as follows: chromatographic column: Rtx-5ms column (30 m × 0.25 mmID × 0.25 μm df). Carrier gas: High-purity helium, carrier gas flow rate: 1.0 mL / min, split ratio: 0.0, gasification temperature of 300°C. Temperature program: initial temperature: 60°C, held for 1.0 min; temperature increased to 120°C at a rate of 10°C·min^-1^, held for 2.0 min; temperature increased to 280°C at a rate of 20°C · min^-1^, held for 4.0 min. Injection volume: 1 μL solvent delay time 2.45 min. Ion source: EI source, electron energy: 70 eV, ion source temperature: 250, interface temperature 250°C, Scanning mass range: m/z 33–600, mass spectrometry search standard library: NIST05, NIST05s and Wiley7 three libraries. The peak acreage of total ion chromatograms (TIC) was applied to determine the relative percentage of GEO compounds.

### Bioassay with major compounds

The compositions of diallyl sulfide and diallyl disulfide in GEO were 8.78% and 30.38%, respectively. Laboratory toxicity tests were carried out by spraying the two compounds in GEO. Diallyl sulfide was purchased from Vander Biotech Co., Ltd. (Beijing, China) and diallyl disulfide was purchased from Yuanye Biotechnology Co., Ltd. (Shanghai, China). The herbicidal activity of the two compounds (diallyl sulfide: 0.01, 0.05, 0.1 and 0.3 g mL^-1^, diallyl disulfide: 0.001, 0.005, 0.01, 0.1 and 0.3 g mL^-1^, 2.5% Tween 80 and 2.5% dimethylformamide, distilled water was added to 10 mL) was evaluated by spraying on barnyard grass. The reagents were mixed well before spraying. A completely randomized design with three replications was applied to all of the experiments. Three untreated pots served as the control and the seedlings weights were recorded after seven days. The fresh weight of barnyard grass seedlings was calculated after 7 days as described above. The seedlings fresh weight inhibition ratio was calculated according to the formula: [1-(experiment group/control group)×100%] [10].

### Defense enzyme activities and chlorophyll content

A GEO mixture of concentration, 0.1 g mL^-1^ (1 g GEO, 0.25 g Tween 80, 0.25 g DMF, distilled water added to 10 mL) was sprayed on barnyard grass seedlings at the three-leaf stage. Fresh samples above the root of seedlings were collected 0, 1, 4, 8, 24, 48 and 72 h after treatment, and stored at -70°C until the activities of defense enzymes (CAT, POD, and SOD) and chlorophyll content were measured. Control samples were collected before the seedlings were treated with the reagents. A completely randomized design with three replications was applied to all of the experiments. The extraction of crude CAT, POD and SOD was done following the instruction of the enzyme assay kit (Nanjing Jiancheng Bioengineering Institute), and according to Gao QT et al with some modifications [14]. The activities of CAT, POD and SOD were measured at 405, 420 and 550 nm using ultraviolet spectrophotometer (A560, AOE Instruments (shanghai) Co., Ltd.), respectively. The results were described as relative activity (%) comparing the last value with the initial one.

The extraction and quantification of chlorophyll a and chlorophyll b were performed by placing 0.2 g of freshly chopped barnyard grass seedlings (greenhouse) treated with GEO into a covered sterile test tube. A volume of 25 mL of decolorizing solution (acetone: ethanol: pure water = 16: 19: 5) was added. The sample was immersed in a dark chamber for 24 hours. Samples were gently agitated three times until the leaf color was completely discolored. Then, the absorbance at 645 and 663 nm was determined using ultraviolet spectrophotometer (A560, AOE Instruments (shanghai) Co., Ltd.). The measured absorbance was used to calculate the corresponding chlorophyll a and chlorophyll b content through the following formulas [15]:

$${\text{C}}_{\text{a}}=\left({\text{A}}_{663}\times 12.72\;-{\text{A}}_{645}\times 2.59\right)\times \text{V}/\left(\text{W}\times 1000\right)$$

$${\text{C}}_{\text{b}}=\left({\text{A}}_{645}\times 22.88-{\text{A}}_{663}\times 4.68\right)\times \text{V}/\left(\text{W}\times 1000\right)$$

### Completely randomized design statistical analyses

The EC_50_ were calculated according to Mardani et al. [16]. Error analysis was calculated using the formula, STDEV.P in Excel 2019. The toxicity regression equation and correlation coefficient in the bioassay were analyzed using the quantitative data machine in data processing system (DPS), and all ANOVA analyses were performed by the single factor statistical analysis method, SNK, in DPS [17]. All bar and line charts were prepared with Origin 75.

## Results

### Inhibition effect of essential oils on growth of barnyard grass seedlings

**Fig 1:**
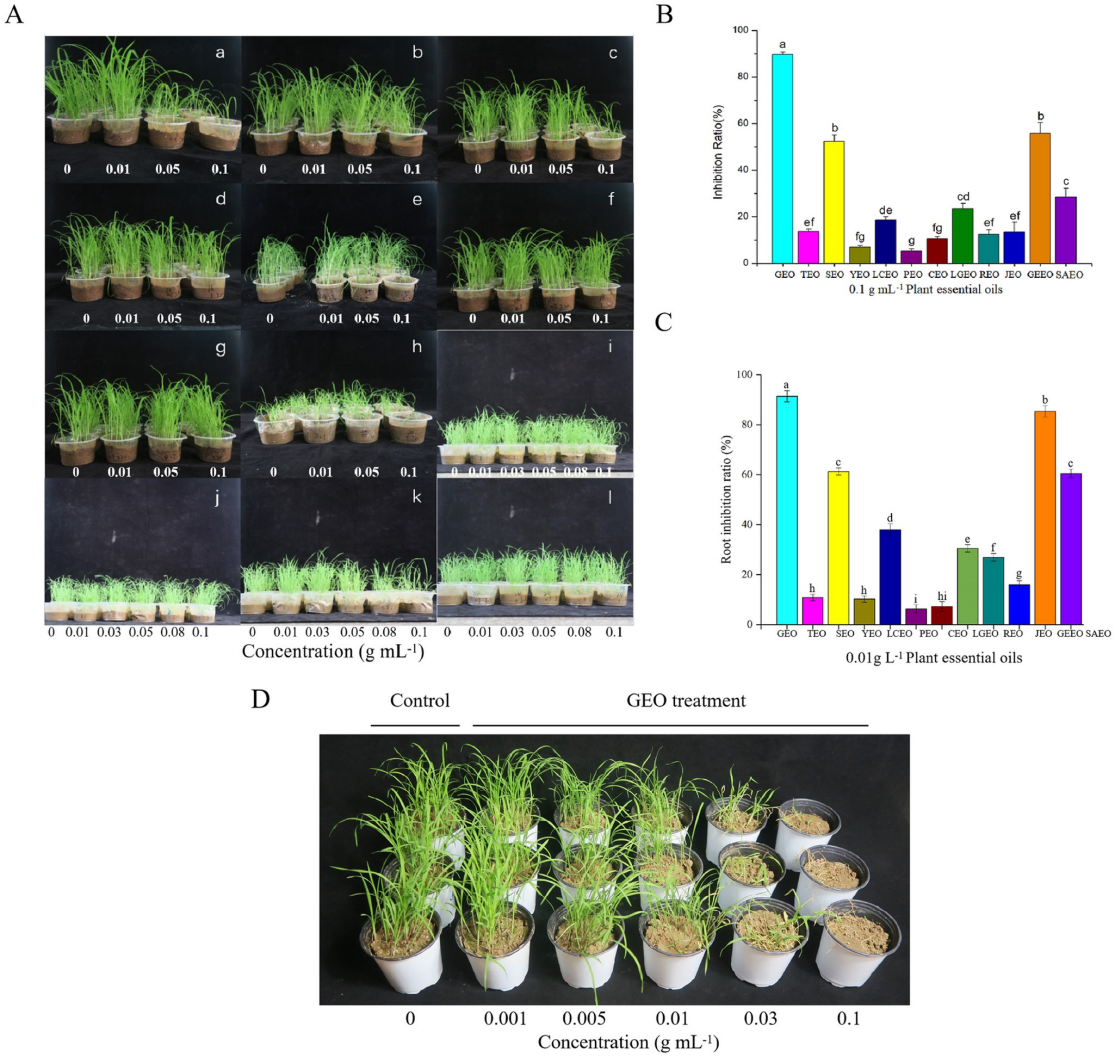
Inhibitory effect of plant essential oils on barnyard grass seedlings. (A) The control effect of twelve essential oils with different concentrations (0, 0.01, 0.03, 0.05, 0.08, 0.1 g mL^-1^) on barnyard grass seedlings. a: Garlic essential oil; b: Tangerine peel essential oil; c: Spearmint essential oil; d: Yellow mugwort essential oil; e: Litsea cubeba essential oil; f: Pine needle essential oil; g: Cedar essential oil; h: Lemon grass essential oil; i: Rosemary essential oil; j: Juniper essential oil; k: Geranium essential oil; l: Star anise essential oil. (B) the average growth inhibition rate of 12 plant essential oils on barnyard grass seedlings after 7 days at a concentration of 0.1 g mL^-1^. Error bars represent the standard error of the mean. Different small letters above the error bars indicate significant differences at 0.05 (ANOVA). The same letters indicate that there were no significant differences among treatments. (GEO: garlic essential oil, TEO: tangerine peel essential oil, SEO: spearmint essential oil, YEO: yellow wormwood leaf essential oil, LCEO: litsea cubeba essential oil, PEO: pine needle essential oil, CEO: cedar essential oil, LGEO: lemon grass essential oil, REO: rosemary essential oil, JEO: juniper essential oil, GEEO: geranium essential oil, SAEO: star anise essential oil). (C) the root length inhibition rate of 12 plant essential oils on barnyard grass seed after 7 days at a concentration of 0.01 g L^-1^. Error bars represent the standard error of the mean. Different small letters above the error bars indicate significant differences at 0.05 (ANOVA). The same letters indicate that there were no significant differences among treatments. (D) the inhibiting effect of GEO on barnyard grass seedlings, the concentrations are 0, 0.001, 0.005, 0.01, 0.03, 0.1 g mL^-1^.

Seven days after treatment, YEO, PEO, CEO, REO, TEO and JEO showed between 5.2 and 13.6% inhibitory effect on barnyard grass (0.1g mL^-1^) (Fig 1A). The inhibition rates of LCEO, LGEO and SAEO at 0.1g mL^-1^ on barnyard grass were 18.7%, 23.53% and 28.43%, respectively (Fig 1B). The inhibition rates of GEO, SEO and GEEO at 0.1g mL^-1^ on barnyard grass were 89.67%, 52.33% and 55.8%, respectively (Fig 1B). When the concentration of essential oil was reduced to 0.05 g mL^-1^, only GEO had an inhibition rate higher than 50% on treated whole plants (Fig 1A). The inhibition rates of GEO, SEO and GEEO at 0.1 g mL^-1^ on the barnyard grass were all more than 50% and the inhibition rate of GEO was 89.67% (Fig 1B).

Root length were used to evaluate inhibitory effect of twelve plant essential oils for barnyard grass. As shown in Fig 1C, the root length inhibition rates of GEO, SEO, GEEO and SAEO at 0.1g L^-1^ on the barnyard grass were all more than 50%. Among them, GEO and GEEO inhibited the root length of barnyard grass by 91.4% and 85.3% respectively. The other eight essential oils showed between 6.3 and 38% root length inhibitory effect on barnyard grass. Please see Fig 1D for the effect of GEO on barnyard grass seedlings. See S1 and S2 Tables for ANOVA tables and comparing means graphs.

### Safety evaluation on rice

**Fig 2:**
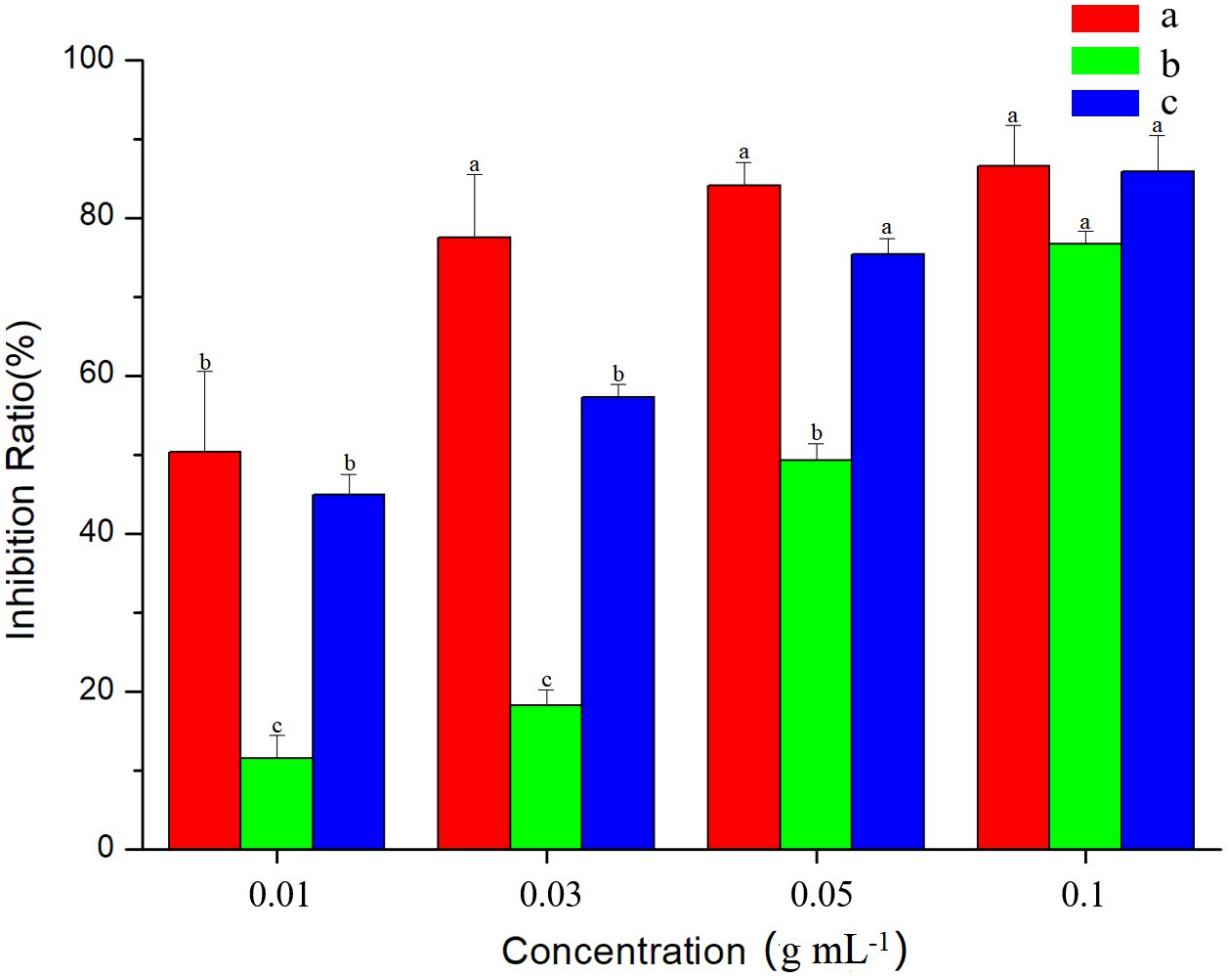
Inhibition effect of GEO on rice. a: seed germination rate; b: inhibition rate of fresh weight at the 1-leaf stage; c: inhibition rate of fresh weight at the 2-leaf stage.

The safety evaluation showed that GEO had inhibitory effects on rice seeds seven days after spraying rice seeds with GEO (Fig 2). The inhibitory effect significantly increased with increase in GEO concentration. The inhibition rate on seed germination was 50% when the concentration of GEO was 0.01 g mL^-1^, and 86.36% when the concentration was 0.1 g mL^-1^.

After 7 days, rice seedlings showed different degrees of withering, an exhibition of the leaf albino phenomenon (S1 Fig). The average inhibitory effect of rice treated with GEO (0.01, 0.03, 0.05, 0.1 g mL^-1^) was 38.89% (1-leaf stage) and 65.86% (2-leaf stage), compared to the control (Table 1). The results indicated that the 1–2 leaf stage of rice seedlings were more tolerant to GEO than the 3-leaf stage of barnyard grass seedlings.

**Table 1:** GEO inhibits rice seedlings growth. The data of fresh weight and inhibition rate of fresh weight in rice treated with GEO (control, 0.01, 0.03, 0.05, 0.1 g mL^-1^).

Concentration (g mL^-1^)	Fresh Weight 1-leaf stage(g)	Fresh Weight 2-leaf stage(g)	1-leaf stage inhibition rate(%)	2-leaf stage inhibition rate(%)	1-leaf stage average inhibition rate(%)	2-leaf stage average inhibition rate(%)
0.01	0.393	0.433	31.771d	55.861b	38.89	65.86
0.03	0.359	0.327	37.674c	66.667ab		
0.05	0.346	0.301	39.931c	69.317a		
0.1	0.310	0.279	46.181bc	71.560a		
control	0.576	0.981	-	-	-	-

### GEO components analysis by GC-MS and its herbicidal activity

**Fig 3:**
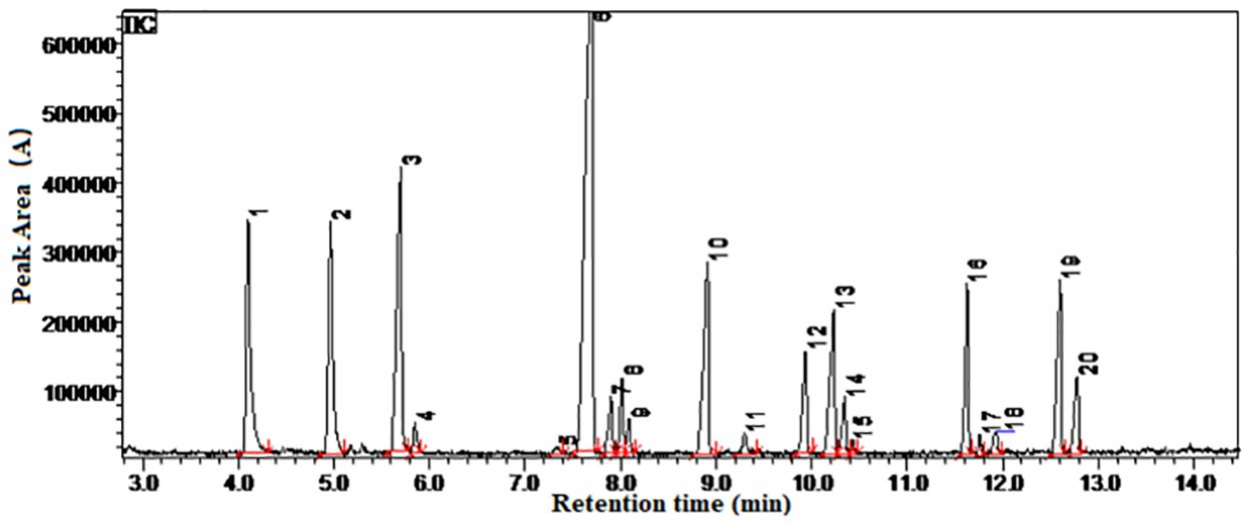
GC-MS chromatogram of the ion peak area of GEO.

**Fig 4:**
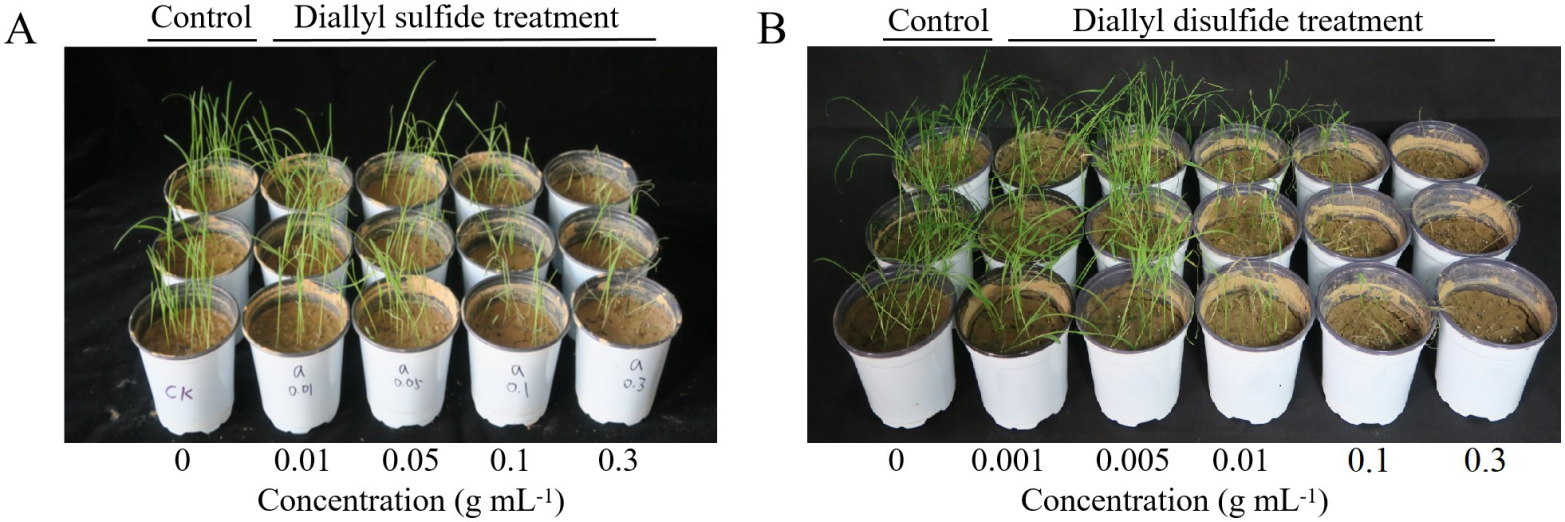
Inhibitory effects of two main compounds with different concentrations on barnyard grass. (A) Diallyl sulfide treatment (0, 0.01, 0.05, 0.1 and 0.3 g mL^-1^); (B) Diallyl disulfide treatment (0, 0.001, 0.005, 0.01, 0.1 and 0.3 g mL^-1^).

GEO was analyzed by gas chromatography-mass spectrometry (Fig 3), and fourteen major chemical components were identified (Table 2). Diallyl disulfide showed better herbicidal effects than diallyl sulfide (Fig 4A and 4B). Bioassay results showed that the EC_50_ values of GEO against barnyard grass seedlings were 0.0126 g mL^-1^ (Table 3). A higher content of two ether compounds (diallyl sulfide and diallyl disulfide) were recorded and their recorded EC_50_ values against barnyard grass seedlings were 0.069 and 0.0153 g mL^-1^, respectively (Table 3).

**Table 2:** GC-MS analysis of the chemical composition of garlic essential oil. (a) no reliable RI data.

Compound	Retention Time	Integration Start Time	End Time of Integration	Peak Area(A)	Peak Area Percentage(A%)	Retention Indices
Diallyl sulfide	4.103	4.000	4.313	1094127	8.78	849
Methyl allyl disulfide	4.967	4.853	5.110	1091824	8.76	a
Ethanethioamide,N,N-dimethyl-	5.699	5.557	5.773	1415789	11.36	1226
Dimethyl trisulfide	5.847	5.803	5.910	116087	0.93	972
1,4-DIMETHOXYBUTANE	7.337	7.263	7.407	42649	0.34	a
Diallyl disulfide	7.700	7.513	7.770	3785620	30.38	1099
Allyl disulfide	7.903	7.813	7.950	224190	1.80	1099
Diallyl disulphide	8.017	7.953	8.047	229038	1.84	1099
1,2,3-thiadiazole,5-Methyl-	8.090	8.047	8.153	115189	0.92	a
Methyl allyl trisulfide	8.910	8.760	9.007	1023967	8.22	a
Thiirane, 2-methyl-	9.300	9.193	9.427	103610	0.83	a
3-Vinyl-3,4-dihydro-1,2-dithiine	9.937	9.830	10.007	414826	3.33	a
Thiazole, 2,4-dimethyl-	10.233	10.110	10.270	699961	5.62	a
3-Vinyl-1,2-dithiacyclohex-5-ene	10.347	10.273	10.393	223258	1.79	1134
Tetrasulfide, dimethyl	10.433	10.400	10.470	40501	0.32	a
Diallyl trisulfide	11.630	11.543	11.670	559543	4.49	1350
Propane,2-isothiocyanato-2-methyl-	11.760	11.717	11.800	56784	0.46	a
Diallyl monosulfide	11.930	11.843	11.983	127320	1.02	1350
dithiolane	12.603	12.497	12.653	741823	5.95	882
1,3-Dithiole-2-thione	12.777	12.700	12.827	356738	2.86	1172

**Table 3:** The relative EC_50_ and regression formula of GEO and its main compounds on barnyard grass.

Compound	Regression formula	Related coefficient	EC_50_ (g mL^-1^)	95% confidence limits	P-value
GEO	Y = 8.38+1.78X	0.9861	0.0126	0.0094~0.0171	0.002
diallyl sulfide	Y = 6.49+1.28X	0.9584	0.069	0.0414~0.1151	0.0416
diallyl disulfide	Y = 7.10+1.16X	0.9904	0.0153	0.0111~0.0212	0.0011

### Defense enzymes activity

**Fig 5:**
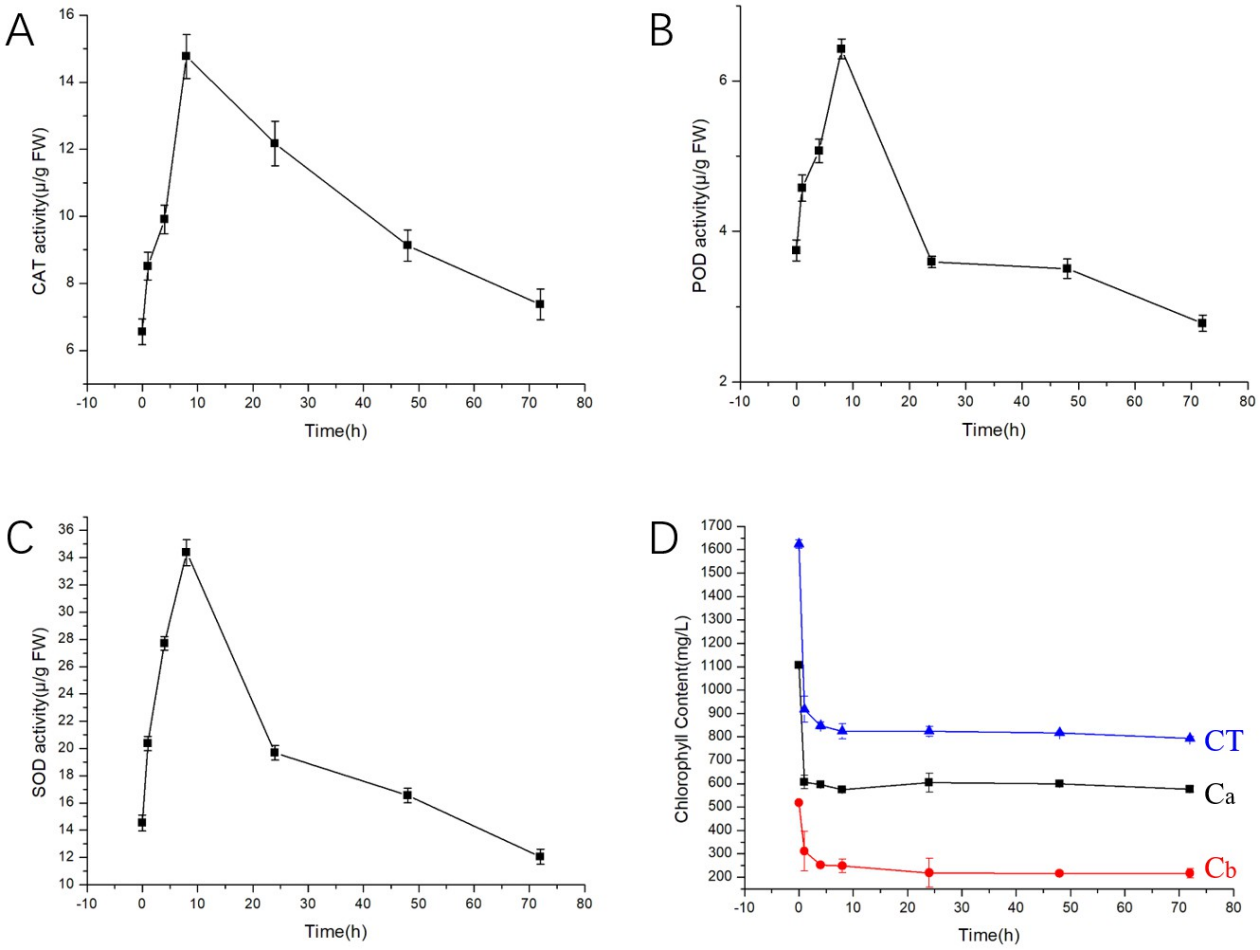
Changes in activities of antioxidant defense enzymes (A-CAT, B-POD, C-SOD) and chlorophyll(D) in barnyard grass seedlings treated with 0.1g mL^-1^ GEO. C_a_: chlorophyll a; C_b_: chlorophyll b; CT: total chlorophyll.

The activities of peroxidase, superoxide dismutase and catalase in barnyard grass treated with GEO increased during the first 8 hours and then decreased after (Fig 5). Chlorophyll a, chlorophyll b and total chlorophyll decreased with increase in time. CAT activity reached a maximum of 14.77 U mgprot^-1^, 8 h after treatment, dropped to a minimum of 7.37 U mgprot^-1^, 72 h after treatment compared to the blank control. The CAT activities increased by 121% (0–8h, compared to control), and decreased (8–72h, compared to the maximum value) by 100%. POD and SOD showed the same trend as CAT, from treatment with GEO. Their maximum enzyme activities were 6.42 U mgprot^-1^ and 34.37 U mgprot^-1^ respectively, but lowered 72 hours after treatment, compared to the blank control. The SOD and POD activities increased by 137% and 110% (0–8h, compared to control), and decreased (8–72h, compared to the maximum value) by 185% and 183%, respectively.

The total chlorophyll content in the control group was 1624.31 μg mg^-1^. Chlorophyll a decreased to 606.26 μg mg^-1^, chlorophyll b decreased to 311.42 μg mg^-1^, and total chlorophyll content decreased to 917.67 μg mg^-1^ one hour after treatment. After 72 hours, chlorophyll a was reduced to 576.59 μg mg^-1^, chlorophyll b to 216.81 μg mg^-1^ and total chlorophyll to only 793.40 μg mg^-1^. In general, the chlorophyll in barnyard grass decreased after spraying GEO.

## Discussion

The bioassay results showed that GEO had a good inhibition effect on barnyard grass. Poonpaiboonpipat et al found that lemongrass essential oil had a significant inhibitory effect on the growth of barnyard grass seedlings [18]. Synowiec et al found that peppermint essential oil and citronella essential oil combined with fatty acid methyl ester had significant effects on the physiology and biochemistry of corn and barnyard grass [19].

The herbicidal effect of plant essential oils can be determined by evaluating their allelopathic components [20]. In our study, we identified two ethers as the main active components of GEO by gas phase mass spectrometry and also found that they had significant inhibitory effect on barnyard grass seedlings. Some essential oil components such as geraniol and carvone, have been reported to inhibit the germination of radish and garden cress seeds [21]. Ether compounds have been widely used in the development of herbicides, such as fomesafen, chlornitrofen, chlomethoxynil, bifenox [22, 23]. Zhao et al designed a diphenyl ether derivative which showed an inhibitory effect on soybean, corn, rice, peanut and cotton [24]. Therefore, the two ether compounds identified in our study may have the potential to be developed as plant-derived herbicides.

The safety of the herbicide on rice was evaluated, as the barnyard grass is the most common malignant weed in rice fields [25]. A previous study showed that the allelochemicals obtained after purification and identification of Welsh onion hot extract, increased the root and stem of rice seedlings by 2.4 times and 1.5 times, respectively, at 1000ppm compared to the control, indicating a significant promoting effect [26]. Ginger and turmeric essential oils was shown to have herbicidal effects, but had no obvious toxicity to tomato, cucumber and rice [27]. In our study we observed that garlic essential oil had inhibitory effect on seed germination and plant growth at various concentrations. A high concentration of GEO affected to some extent the growth of rice seedlings, while a low concentration showed less inhibitory effect on the growth of rice seedlings.

To preliminarily explore the mechanism of inhibition, enzyme activity experiments were carried out. CAT, POD, and SOD are antioxidant enzyme protection systems [28], which play vital roles in the maintenance of a dynamic equilibrium state in plants under stress [29]. Our results showed that CAT, POD and SOD activities all increased for 8 hours and then decreased after treatment with GEO. This stress-induced variation in enzyme activities is a typical response of plants under stressed environments [29], such as high-temperature stress [30], metallic stress [31], and salt stress [32]. The persistence of the stress and increase in peroxide accumulation, cause damage to cells, which may result in death [33]. Chlorophyll content directly reflects the growth status and photosynthetic capacity of plants [34]. Both chlorophyll a and b showed a decreasing trend after GEO treatment at the same concentration, i.e., the total chlorophyll content in barnyard grass decreased with increase in time. The results showed that the chloroplast of barnyard grass was damaged after application of high concentration of GEO, resulting in the inhibition of photosynthesis [35]. GEO therefore effectively inhibited the growth and development of barnyard grass seedlings, by inhibiting the antioxidant enzyme system of barnyard grass and reducing chlorophyll content which inhibited photosynthesis.

## Conclusions

Evaluation of twelve different plant essential oils showed that GEO had the strongest inhibition effect on the growth of barnyard grass seedlings. It also significantly inhibited the germination of rice seeds, and also the growth of rice seedlings at the first and second leaf stages to varying degrees. This indicated that GEO may be risky for rice fields.

Barnyard grass seedlings treated with GEO of varying concentration (0.01–0.1 g mL^-1^), showed dose-dependent death and bleaching. The activities of CAT, POD and SOD enzymes increased after 8 hours and then decreased. GC-MS analysis detected 14 major compounds in GEO. The major compounds (diallyl sulfide and diallyl disulfide) had herbicidal effects and can be developed as new herbicides for the control of barnyard grass.

## Supporting information

S1 Fig Effects of GEO on rice seeds and seedlings. (A) the germination of rice seeds after spraying with GEO (0.01, 0.03, 0.05, 0.1 g mL^-1^). (B, C) the efficacy of spraying GEO on rice seedlings at the 1, 2-leaf stage. CK, 1, 2, 3, 4 were 0, 0.01, 0.03, 0.05, 0.1 g mL^-1^.
(TIF)

S1 Table ANOVA table. (DOCX)

S2 Table Comparing means. (DOCX)

S1 File (DOC)

S1 Data (XLSX)

S2 Data (XLSX)

S3 Data (XLSX)
