# Supplementary figures and images for: Phytochemical profiling and allelopathic effect of garlic essential oil on barnyard grass (Echinochloa crusgalli L.)
Source: PLoS One. 2023 Apr 25;18(4):e0272842. doi: 10.1371/journal.pone.0272842 (PMC10128991; doi:10.1371/journal.pone.0272842)

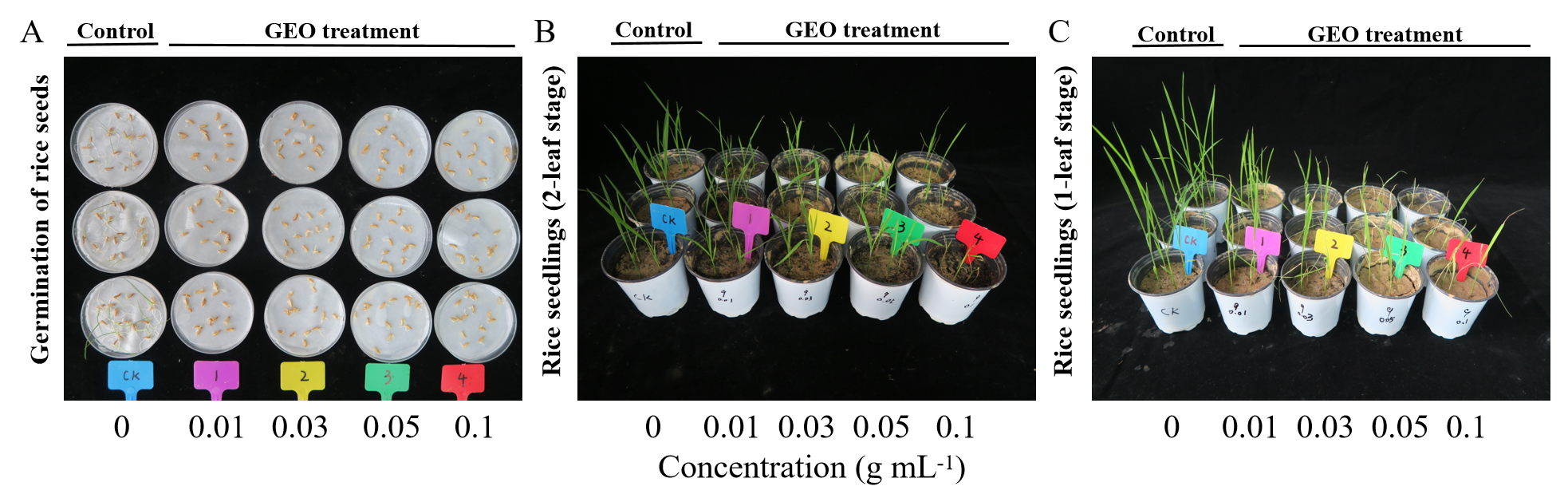

Supplement: S1 Fig — (A) the germination of rice seeds after spraying with GEO (0.01, 0.03, 0.05, 0.1 g mL-1). (B, C) the efficacy of spraying GEO on rice seedlings at the 1, 2-leaf stage. CK, 1, 2, 3, 4 were 0, 0.01, 0.03, 0.05, 0.1 g mL-1. (TIF) [file pone.0272842.s001.tif]
