# Supplementary material for: Phytochemical profiling and allelopathic effect of garlic essential oil on barnyard grass (Echinochloa crusgalli L.)
Source: PLoS One. 2023 Apr 25;18(4):e0272842. doi: 10.1371/journal.pone.0272842 (PMC10128991; doi:10.1371/journal.pone.0272842)
Supplement: S1 Table — (DOCX) [file pone.0272842.s002.docx]

**Table S1.** ANOVA table.

| Barnyard grass seedling at 0.01g mL^-1^ treatment ANOVA | | | | | | |
| --- | --- | --- | --- | --- | --- | --- |
| EOs | sample size | average | standard deviation | standard error | 95% confidence interval | |
| GEO | 3 | 43.7667 | 1.4048 | 0.8110 | 40.2771 | 47.2563 |
| TEO | 3 | 5.0333 | 1.3868 | 0.8007 | 1.5882 | 8.4784 |
| SEO | 3 | 13.4333 | 1.5177 | 0.8762 | 9.6632 | 17.2034 |
| YEO | 3 | 4.4667 | 0.9452 | 0.5457 | 2.1188 | 6.8146 |
| LCEO | 3 | 7.7333 | 1.4048 | 0.8110 | 4.2437 | 11.2229 |
| PEO | 3 | 3.0333 | 1.2014 | 0.6936 | 0.0489 | 6.0177 |
| CEO | 3 | 4.7667 | 1.2897 | 0.7446 | 1.5629 | 7.9705 |
| LGEO | 3 | 8.4667 | 0.7024 | 0.4055 | 6.7219 | 10.2115 |
| REO | 3 | 5.0667 | 0.7767 | 0.4485 | 3.1371 | 6.9962 |
| JEO | 3 | 6.5333 | 0.7506 | 0.4333 | 4.6689 | 8.3978 |
| GEEO | 3 | 16.9333 | 1.5535 | 0.8969 | 13.0742 | 20.7924 |
| SAEO | 3 | 10.4333 | 0.6028 | 0.3480 | 8.9360 | 11.9307 |

| Barnyard grass seedling at 0.01g mL^-1^ treatment ANOVA table | | | | | |
| --- | --- | --- | --- | --- | --- |
| Source | Sum of Squares | Degree of freedom | Mean square | F-value | P-value |
| Between treatments | 4102.4989 | 11 | 372.9544 | 269.4430 | 0.0001 |
| In treatments | 33.2200 | 24 | 1.3842 |  |  |
| Total variation | 4135.7189 | 35 |  |  |  |

| Barnyard grass seedling at 0.03g mL^-1^ treatment ANOVA | | | | | | |
| --- | --- | --- | --- | --- | --- | --- |
| EOs | sample size | average | standard deviation | standard error | 95% confidence interval | |
| REO | 3 | 7.6000 | 0.8544 | 0.4933 | 5.4776 | 9.7224 |
| JEO | 3 | 9.3333 | 0.6506 | 0.3756 | 7.7171 | 10.9496 |
| GEEO | 3 | 27.6333 | 1.1676 | 0.6741 | 24.7328 | 30.5339 |
| SAEO | 3 | 15.4333 | 1.2055 | 0.6960 | 12.4386 | 18.4281 |

| Barnyard grass seedling at 0.03g mL^-1^ treatment ANOVA table | | | | | |
| --- | --- | --- | --- | --- | --- |
| Source | Sum of Squares | Degree of freedom | Mean square | F-value | P-value |
| Between treatments | 739.9800 | 3 | 246.6600 | 248.5240 | 0.0001 |
| In treatments | 7.9400 | 8 | 0.9925 |  |  |
| Total variation | 747.9200 | 11 |  |  |  |

| Barnyard grass seedling at 0.05g mL^-1^ treatment ANOVA | | | | | | |
| --- | --- | --- | --- | --- | --- | --- |
| EOs | sample size | average | standard deviation | standard error | 95% confidence interval | |
| GEO | 3 | 68.7000 | 1.6093 | 0.9292 | 64.7022 | 72.6978 |
| TEO | 3 | 9.2667 | 1.0504 | 0.6064 | 6.6573 | 11.8760 |
| SEO | 3 | 37.8667 | 1.5631 | 0.9025 | 33.9837 | 41.7497 |
| YEO | 3 | 6.1000 | 0.6245 | 0.3606 | 4.5487 | 7.6513 |
| LCEO | 3 | 10.8333 | 1.3051 | 0.7535 | 7.5912 | 14.0754 |
| PEO | 3 | 3.9333 | 1.1060 | 0.6386 | 1.1858 | 6.6809 |
| CEO | 3 | 6.7667 | 1.0066 | 0.5812 | 4.2660 | 9.2673 |
| LGEO | 3 | 13.9333 | 1.3650 | 0.7881 | 10.5424 | 17.3243 |
| REO | 3 | 9.0667 | 1.3204 | 0.7623 | 5.7867 | 12.3466 |
| JEO | 3 | 10.4333 | 1.9553 | 1.1289 | 5.5760 | 15.2907 |
| GEEO | 3 | 41.1000 | 1.5875 | 0.9165 | 37.1566 | 45.0434 |
| SAEO | 3 | 23.8667 | 1.6258 | 0.9387 | 19.8279 | 27.9055 |

| Barnyard grass seedling at 0.05g mL^-1^ treatment ANOVA table | | | | | |
| --- | --- | --- | --- | --- | --- |
| Source | Sum of Squares | Degree of freedom | Mean square | F-value | P-value |
| Between treatments | 12673.0288 | 11 | 1152.0935 | 599.5280 | 0.0001 |
| In treatments | 46.1200 | 24 | 1.9217 |  |  |
| Total variation | 12719.1488 | 35 |  |  |  |

| Barnyard grass seedling at 0.08g mL^-1^ treatment ANOVA | | | | | | |
| --- | --- | --- | --- | --- | --- | --- |
| EOs | sample size | average | standard deviation | standard error | 95% confidence interval | |
| REO | 3 | 11.3333 | 1.6166 | 0.9333 | 7.3175 | 15.3491 |
| JEO | 3 | 11.9333 | 1.7616 | 1.0171 | 7.5572 | 16.3095 |
| GEEO | 3 | 45.0000 | 3.3511 | 1.9348 | 36.6754 | 53.3246 |
| SAEO | 3 | 25.8333 | 1.8610 | 1.0745 | 21.2103 | 30.4563 |

| Barnyard grass seedling at 0.08g mL^-1^ treatment ANOVA table | | | | | |
| --- | --- | --- | --- | --- | --- |
| Source | Sum of Squares | Degree of freedom | Mean square | F-value | P-value |
| Between treatments | 2248.5224 | 3 | 749.5075 | 146.8900 | 0.0001 |
| In treatments | 40.8200 | 8 | 5.1025 |  |  |
| Total variation | 2289.3424 | 11 |  |  |  |

| Barnyard grass seedling at 0.1g mL^-1^ treatment ANOVA | | | | | | |
| --- | --- | --- | --- | --- | --- | --- |
| EOs | sample size | average | standard deviation | standard error | 95% confidence interval | |
| GEO | 3 | 89.6667 | 1.3051 | 0.7535 | 86.4246 | 92.9088 |
| TEO | 3 | 13.6 | 1.4526 | 0.8386 | 9.9916 | 17.2084 |
| SEO | 3 | 52.3333 | 3.4269 | 1.9785 | 43.8206 | 60.8461 |
| YEO | 3 | 6.9 | 0.8888 | 0.5132 | 4.692 | 9.108 |
| LCEO | 3 | 18.7 | 1.6643 | 0.9609 | 14.5656 | 22.8344 |
| PEO | 3 | 5.3333 | 1.3051 | 0.7535 | 2.0912 | 8.5754 |
| CEO | 3 | 10.7667 | 0.8505 | 0.491 | 8.6539 | 12.8794 |
| LGEO | 3 | 23.5333 | 2.761 | 1.5941 | 16.6745 | 30.3921 |
| REO | 3 | 12.6 | 2.3431 | 1.3528 | 6.7795 | 18.4205 |
| JEO | 3 | 13.4333 | 5.1394 | 2.9672 | 0.6664 | 26.2003 |
| GEEO | 3 | 55.8 | 5.5018 | 3.1765 | 42.1327 | 69.4673 |
| SAEO | 3 | 28.4333 | 4.7248 | 2.7278 | 16.6964 | 40.1703 |

| Barnyard grass seedling at 0.1g mL^-1^ treatment ANOVA table | | | | | |
| --- | --- | --- | --- | --- | --- |
| Source | Sum of Squares | Degree of freedom | Mean square | F-value | P-value |
| Between treatments | 4102.4989 | 11 | 372.9544 | 269.4430 | 0.0001 |
| In treatments | 33.2200 | 24 | 1.3842 |  |  |
| Total variation | 4135.7189 | 35 |  |  |  |

| Barnyard grass root length at 100 ppm treatment ANOVA | | | | | | |
| --- | --- | --- | --- | --- | --- | --- |
| EOs | sample size | average | standard deviation | standard error | 95% confidence interval | |
| GEO | 3 | 91.4000 | 2.2517 | 1.3000 | 85.8066 | 96.9934 |
| TEO | 3 | 10.8333 | 1.3051 | 0.7535 | 7.5912 | 14.0754 |
| SEO | 3 | 61.2667 | 1.4503 | 0.8373 | 57.6640 | 64.8694 |
| YEO | 3 | 10.2000 | 1.2530 | 0.7234 | 7.0874 | 13.3126 |
| LCEO | 3 | 37.9667 | 2.4583 | 1.4193 | 31.8599 | 44.0735 |
| PEO | 3 | 6.3333 | 1.7039 | 0.9838 | 2.1006 | 10.5661 |
| CEO | 3 | 7.2667 | 1.8230 | 1.0525 | 2.7381 | 11.7953 |
| LGEO | 3 | 30.5333 | 1.4640 | 0.8452 | 26.8965 | 34.1701 |
| REO | 3 | 26.9667 | 1.5044 | 0.8686 | 23.2294 | 30.7039 |
| JEO | 3 | 15.9667 | 1.6166 | 0.9333 | 11.9509 | 19.9825 |
| GEEO | 3 | 85.3333 | 2.3029 | 1.3296 | 79.6126 | 91.0540 |
| SAEO | 3 | 60.5000 | 1.7521 | 1.0116 | 56.1474 | 64.8526 |

| Barnyard grass root length at 100 ppm treatment ANOVA table | | | | | |
| --- | --- | --- | --- | --- | --- |
| Source | Sum of Squares | Degree of freedom | Mean square | F-value | P-value |
| Between treatments | 30749.5101 | 11 | 2795.4100 | 880.2130 | 0.0001 |
| In treatments | 76.2200 | 24 | 3.1758 |  |  |
| Total variation | 30825.7301 | 35 |  |  |  |
