# Supplementary material for: Phytochemical profiling and allelopathic effect of garlic essential oil on barnyard grass (Echinochloa crusgalli L.)
Source: PLoS One. 2023 Apr 25;18(4):e0272842. doi: 10.1371/journal.pone.0272842 (PMC10128991; doi:10.1371/journal.pone.0272842)
Supplement: S2 Table — (DOCX) [file pone.0272842.s003.docx]

| Barnyard grass seedling at 0.01g mL^-1^ treatment SNK multiple comparison | | | | | | | | | | | | | |
| --- | --- | --- | --- | --- | --- | --- | --- | --- | --- | --- | --- | --- | --- |
| EOs | average | GEO | GEEO | SEO | SAEO | LGEO | LCEO | JEO | REO | TEO | CEO | YEO | PEO |
| GEO | 43.7667 |  | 0.0000 | 0.0000 | 0.0000 | 0.0000 | 0.0000 | 0.0000 | 0.0000 | 0.0000 | 0.0000 | 0.0000 | 0.0000 |
| GEEO | 16.9333 | 26.8333 |  | 0.0013 | 0.0000 | 0.0000 | 0.0000 | 0.0000 | 0.0000 | 0.0000 | 0.0000 | 0.0000 | 0.0000 |
| SEO | 13.4333 | 30.3333 | 3.5000 |  | 0.0046 | 0.0001 | 0.0000 | 0.0000 | 0.0000 | 0.0000 | 0.0000 | 0.0000 | 0.0000 |
| SAEO | 10.4333 | 33.3333 | 6.5000 | 3.0000 |  | 0.0517 | 0.0253 | 0.0024 | 0.0001 | 0.0001 | 0.0001 | 0.0000 | 0.0000 |
| LGEO | 8.4667 | 35.3000 | 8.4667 | 4.9667 | 1.9667 |  | 0.4527 | 0.1308 | 0.0085 | 0.0121 | 0.0088 | 0.0056 | 0.0002 |
| LCEO | 7.7333 | 36.0333 | 9.2000 | 5.7000 | 2.7000 | 0.7333 |  | 0.2236 | 0.0273 | 0.0447 | 0.0367 | 0.0253 | 0.0009 |
| JEO | 6.5333 | 37.2333 | 10.4000 | 6.9000 | 3.9000 | 1.9333 | 1.2000 |  | 0.1399 | 0.2813 | 0.2804 | 0.2320 | 0.0144 |
| REO | 5.0667 | 38.7000 | 11.8667 | 8.3667 | 5.3667 | 3.4000 | 2.6667 | 1.4667 |  | 0.9726 | 0.9478 | 0.9232 | 0.2458 |
| TEO | 5.0333 | 38.7333 | 11.9000 | 8.4000 | 5.4000 | 3.4333 | 2.7000 | 1.5000 | 0.0333 |  | 0.7837 | 0.8268 | 0.1876 |
| CEO | 4.7667 | 39.0000 | 12.1667 | 8.6667 | 5.6667 | 3.7000 | 2.9667 | 1.7667 | 0.3000 | 0.2667 |  | 0.7575 | 0.1895 |
| YEO | 4.4667 | 39.3000 | 12.4667 | 8.9667 | 5.9667 | 4.0000 | 3.2667 | 2.0667 | 0.6000 | 0.5667 | 0.3000 |  | 0.1487 |
| PEO | 3.0333 | 40.7333 | 13.9000 | 10.4000 | 7.4000 | 5.4333 | 4.7000 | 3.5000 | 2.0333 | 2.0000 | 1.7333 | 1.4333 |  |

**Table S2.** Comparing means.

*lower triangle is mean difference, upper triangle is significance level

| Barnyard grass seedling at 0.01g mL^-1^ treatment SNK multiple comparison result | | | | |
| --- | --- | --- | --- | --- |
| EOs | average | 5%significant leve |  | 1%Extreme significance level |
| GEO | 43.7667 | a |  | A |
| GEEO | 16.9333 | b |  | B |
| SEO | 13.4333 | c |  | C |
| SAEO | 10.4333 | d |  | D |
| LGEO | 8.4667 | de |  | DE |
| LCEO | 7.7333 | e |  | DEF |
| JEO | 6.5333 | ef |  | EFG |
| REO | 5.0667 | fg |  | EFG |
| TEO | 5.0333 | fg |  | EFG |
| CEO | 4.7667 | fg |  | FG |
| YEO | 4.4667 | fg |  | FG |
| PEO | 3.0333 | g |  | G |

| Barnyard grass seedling at 0.03g mL^-1^ treatment SNK multiple comparison | | | | | |
| --- | --- | --- | --- | --- | --- |
| EOs | average | GEEO | SAEO | JEO | REO |
| GEEO | 27.6333 |  | 0.0000 | 0.0000 | 0.0000 |
| SAEO | 15.4333 | 12.2000 |  | 0.0001 | 0.0000 |
| JEO | 9.3333 | 18.3000 | 6.1000 |  | 0.0657 |
| REO | 7.6000 | 20.0333 | 7.8333 | 1.7333 |  |

*lower triangle is mean difference, upper triangle is significance level

| Barnyard grass seedling at 0.03g mL^-1^ treatment SNK multiple comparison result | | | | |
| --- | --- | --- | --- | --- |
| EOs | average | 5%significant leve |  | 1%Extreme significance level |
| GEEO | 27.6333 | a |  | A |
| SAEO | 15.4333 | b |  | B |
| JEO | 9.3333 | c |  | C |
| REO | 7.6000 | c |  | C |

| Barnyard grass seedling at 0.05g mL^-1^ treatment SNK multiple comparison | | | | | | | | | | | | | |
| --- | --- | --- | --- | --- | --- | --- | --- | --- | --- | --- | --- | --- | --- |
| EOs | average | GEO | GEEO | SEO | SAEO | LGEO | LCEO | JEO | TEO | REO | CEO | YEO | PEO |
| GEO | 68.7000 |  | 0.0000 | 0.0000 | 0.0000 | 0.0000 | 0.0000 | 0.0000 | 0.0000 | 0.0000 | 0.0000 | 0.0000 | 0.0000 |
| GEEO | 41.1000 | 27.6000 |  | 0.0087 | 0.0000 | 0.0000 | 0.0000 | 0.0000 | 0.0000 | 0.0000 | 0.0000 | 0.0000 | 0.0000 |
| SEO | 37.8667 | 30.8333 | 3.2333 |  | 0.0000 | 0.0000 | 0.0000 | 0.0000 | 0.0000 | 0.0000 | 0.0000 | 0.0000 | 0.0000 |
| SAEO | 23.8667 | 44.8333 | 17.2333 | 14.0000 |  | 0.0000 | 0.0000 | 0.0000 | 0.0000 | 0.0000 | 0.0000 | 0.0000 | 0.0000 |
| LGEO | 13.9333 | 54.7667 | 27.1667 | 23.9333 | 9.9333 |  | 0.0114 | 0.0133 | 0.0020 | 0.0021 | 0.0000 | 0.0000 | 0.0000 |
| LCEO | 10.8333 | 57.8667 | 30.2667 | 27.0333 | 13.0333 | 3.1000 |  | 0.7269 | 0.3648 | 0.4189 | 0.0116 | 0.0040 | 0.0001 |
| JEO | 10.4333 | 58.2667 | 30.6667 | 27.4333 | 13.4333 | 3.5000 | 0.4000 |  | 0.3129 | 0.4604 | 0.0171 | 0.0066 | 0.0001 |
| TEO | 9.2667 | 59.4333 | 31.8333 | 28.6000 | 14.6000 | 4.6667 | 1.5667 | 1.1667 |  | 0.8612 | 0.0900 | 0.0460 | 0.0008 |
| REO | 9.0667 | 59.6333 | 32.0333 | 28.8000 | 14.8000 | 4.8667 | 1.7667 | 1.3667 | 0.2000 |  | 0.0534 | 0.0384 | 0.0007 |
| CEO | 6.7667 | 61.9333 | 34.3333 | 31.1000 | 17.1000 | 7.1667 | 4.0667 | 3.6667 | 2.5000 | 2.3000 |  | 0.5614 | 0.0494 |
| YEO | 6.1000 | 62.6000 | 35.0000 | 31.7667 | 17.7667 | 7.8333 | 4.7333 | 4.3333 | 3.1667 | 2.9667 | 0.6667 |  | 0.0676 |
| PEO | 3.9333 | 64.7667 | 37.1667 | 33.9333 | 19.9333 | 10.0000 | 6.9000 | 6.5000 | 5.3333 | 5.1333 | 2.8333 | 2.1667 |  |

*lower triangle is mean difference, upper triangle is significance level

| Barnyard grass seedling at 0.05g mL^-1^ treatment SNK multiple comparison result | | | | |
| --- | --- | --- | --- | --- |
| EOs | average | 5%significant leve |  | 1%Extreme significance level |
| GEO | 68.7000 | a |  | A |
| GEEO | 41.1000 | b |  | B |
| SEO | 37.8667 | c |  | C |
| SAEO | 23.8667 | d |  | D |
| LGEO | 13.9333 | e |  | E |
| LCEO | 10.8333 | f |  | EF |
| JEO | 10.4333 | f |  | EF |
| TEO | 9.2667 | fg |  | FG |
| REO | 9.0667 | fg |  | FG |
| CEO | 6.7667 | gh |  | FGH |
| YEO | 6.1000 | hi |  | GH |
| PEO | 3.9333 | i |  | H |

| Barnyard grass seedling at 0.08g mL^-1^ treatment SNK multiple comparison | | | | | |
| --- | --- | --- | --- | --- | --- |
| EOs | average | GEEO | SAEO | JEO | REO |
| GEEO | 45.0000 |  | 0.0000 | 0.0000 | 0.0000 |
| SAEO | 25.8333 | 19.1667 |  | 0.0001 | 0.0001 |
| JEO | 11.9333 | 33.0667 | 13.9000 |  | 0.7533 |
| REO | 11.3333 | 33.6667 | 14.5000 | 0.6000 |  |

*lower triangle is mean difference, upper triangle is significance level

| Barnyard grass seedling at 0.08g mL^-1^ treatment SNK multiple comparison result | | | | |
| --- | --- | --- | --- | --- |
| EOs | average | 5%significant leve |  | 1%Extreme significance level |
| GEEO | 45.0000 | a |  | A |
| SAEO | 25.8333 | b |  | B |
| JEO | 11.9333 | c |  | C |
| REO | 11.3333 | c |  | C |

| Barnyard grass seedling at 0.1g mL^-1^ treatment SNK multiple comparison | | | | | | | | | | | | | |
| --- | --- | --- | --- | --- | --- | --- | --- | --- | --- | --- | --- | --- | --- |
| EOs | average | GEO | GEEO | SEO | SAEO | LGEO | LCEO | TEO | JEO | REO | CEO | YEO | PEO |
| GEO | 89.6667 |  | 0 | 0 | 0 | 0 | 0 | 0 | 0 | 0 | 0 | 0 | 0 |
| GEEO | 55.8 | 33.8667 |  | 0.1804 | 0 | 0 | 0 | 0 | 0 | 0 | 0 | 0 | 0 |
| SEO | 52.3333 | 37.3333 | 3.4667 |  | 0 | 0 | 0 | 0 | 0 | 0 | 0 | 0 | 0 |
| SAEO | 28.4333 | 61.2333 | 27.3667 | 23.9 |  | 0.063 | 0.002 | 0 | 0 | 0 | 0 | 0 | 0 |
| LGEO | 23.5333 | 66.1333 | 32.2667 | 28.8 | 4.9 |  | 0.0664 | 0.0017 | 0.0026 | 0.0018 | 0.0004 | 0 | 0 |
| LCEO | 18.7 | 70.9667 | 37.1 | 33.6333 | 9.7333 | 4.8333 |  | 0.0536 | 0.1119 | 0.0987 | 0.0315 | 0.0011 | 0.0003 |
| TEO | 13.6 | 76.0667 | 42.2 | 38.7333 | 14.8333 | 9.9333 | 5.1 |  | 0.9477 | 0.9167 | 0.6765 | 0.0895 | 0.0324 |
| JEO | 13.4333 | 76.2333 | 42.3667 | 38.9 | 15 | 10.1 | 5.2667 | 0.1667 |  | 0.7431 | 0.5467 | 0.0697 | 0.0272 |
| REO | 12.6 | 77.0667 | 43.2 | 39.7333 | 15.8333 | 10.9333 | 6.1 | 1 | 0.8333 |  | 0.4727 | 0.08 | 0.0375 |
| CEO | 10.7667 | 78.9 | 45.0333 | 41.5667 | 17.6667 | 12.7667 | 7.9333 | 2.8333 | 2.6667 | 1.8333 |  | 0.137 | 0.0986 |
| YEO | 6.9 | 82.7667 | 48.9 | 45.4333 | 21.5333 | 16.6333 | 11.8 | 6.7 | 6.5333 | 5.7 | 3.8667 |  | 0.5389 |
| PEO | 5.3333 | 84.3333 | 50.4667 | 47 | 23.1 | 18.2 | 13.3667 | 8.2667 | 8.1 | 7.2667 | 5.4333 | 1.5667 |  |

*lower triangle is mean difference, upper triangle is significance level

| Barnyard grass seedling at 0.1g mL^-1^ treatment SNK multiple comparison result | | | | |
| --- | --- | --- | --- | --- |
| EOs | average | 5%significant leve |  | 1%Extreme significance level |
| GEO | 89.6667 | a |  | A |
| GEEO | 55.8 | b |  | B |
| SEO | 52.3333 | b |  | B |
| SAEO | 28.4333 | c |  | C |
| LGEO | 23.5333 | cd |  | CD |
| LCEO | 18.7 | de |  | DE |
| TEO | 13.6 | ef |  | EF |
| JEO | 13.4333 | ef |  | EF |
| REO | 12.6 | ef |  | EF |
| CEO | 10.7667 | fg |  | EF |
| YEO | 6.9 | fg |  | F |
| PEO | 5.3333 | g |  | F |

| Barnyard grass root length at 0.1g mL^-1^ treatment SNK multiple comparison | | | | | | | | | | | | | |
| --- | --- | --- | --- | --- | --- | --- | --- | --- | --- | --- | --- | --- | --- |
| EOs | average | GEO | GEEO | SEO | SAEO | LCEO | LGEO | REO | JEO | TEO | YEO | CEO | PEO |
| GEO | 91.4000 |  | 0.0003 | 0.0000 | 0.0000 | 0.0000 | 0.0000 | 0.0000 | 0.0000 | 0.0000 | 0.0000 | 0.0000 | 0.0000 |
| GEEO | 85.3333 | 6.0667 |  | 0.0000 | 0.0000 | 0.0000 | 0.0000 | 0.0000 | 0.0000 | 0.0000 | 0.0000 | 0.0000 | 0.0000 |
| SEO | 61.2667 | 30.1333 | 24.0667 |  | 0.6031 | 0.0000 | 0.0000 | 0.0000 | 0.0000 | 0.0000 | 0.0000 | 0.0000 | 0.0000 |
| SAEO | 60.5000 | 30.9000 | 24.8333 | 0.7667 |  | 0.0000 | 0.0000 | 0.0000 | 0.0000 | 0.0000 | 0.0000 | 0.0000 | 0.0000 |
| LCEO | 37.9667 | 53.4333 | 47.3667 | 23.3000 | 22.5333 |  | 0.0000 | 0.0000 | 0.0000 | 0.0000 | 0.0000 | 0.0000 | 0.0000 |
| LGEO | 30.5333 | 60.8667 | 54.8000 | 30.7333 | 29.9667 | 7.4333 |  | 0.0219 | 0.0000 | 0.0000 | 0.0000 | 0.0000 | 0.0000 |
| REO | 26.9667 | 64.4333 | 58.3667 | 34.3000 | 33.5333 | 11.0000 | 3.5667 |  | 0.0000 | 0.0000 | 0.0000 | 0.0000 | 0.0000 |
| JEO | 15.9667 | 75.4333 | 69.3667 | 45.3000 | 44.5333 | 22.0000 | 14.5667 | 11.0000 |  | 0.0017 | 0.0016 | 0.0000 | 0.0000 |
| TEO | 10.8333 | 80.5667 | 74.5000 | 50.4333 | 49.6667 | 27.1333 | 19.7000 | 16.1333 | 5.1333 |  | 0.6673 | 0.0551 | 0.0240 |
| YEO | 10.2000 | 81.2000 | 75.1333 | 51.0667 | 50.3000 | 27.7667 | 20.3333 | 16.7667 | 5.7667 | 0.6333 |  | 0.0551 | 0.0355 |
| CEO | 7.2667 | 84.1333 | 78.0667 | 54.0000 | 53.2333 | 30.7000 | 23.2667 | 19.7000 | 8.7000 | 3.5667 | 2.9333 |  | 0.5273 |
| PEO | 6.3333 | 85.0667 | 79.0000 | 54.9333 | 54.1667 | 31.6333 | 24.2000 | 20.6333 | 9.6333 | 4.5000 | 3.8667 | 0.9333 |  |

*lower triangle is mean difference, upper triangle is significance level

| Barnyard grass root length at 0.1g mL^-1^ treatment SNK multiple comparison result | | | | |
| --- | --- | --- | --- | --- |
| EOs | average | 5%significant leve |  | 1%Extreme significance level |
| GEO | 91.4000 | a |  | A |
| GEEO | 85.3333 | b |  | B |
| SEO | 61.2667 | c |  | C |
| SAEO | 60.5000 | c |  | C |
| LCEO | 37.9667 | d |  | D |
| LGEO | 30.5333 | e |  | E |
| REO | 26.9667 | f |  | E |
| JEO | 15.9667 | g |  | F |
| TEO | 10.8333 | h |  | G |
| YEO | 10.2000 | h |  | G |
| CEO | 7.2667 | hi |  | G |
| PEO | 6.3333 | i |  | G |
