# Supplementary material for: Phytochemical profiling and allelopathic effect of garlic essential oil on barnyard grass (Echinochloa crusgalli L.)
Source: PLoS One. 2023 Apr 25;18(4):e0272842. doi: 10.1371/journal.pone.0272842 (PMC10128991; doi:10.1371/journal.pone.0272842)
Supplement: S1 File — (DOC) [file pone.0272842.s004.doc]

GEO

**GC-MS analysis of the chemical composition of garlic essential oil**

| Compound | Retention Time | Integration Start Time | End Time of Integration | Peak Area（A） | Peak Area Percentage（A%） | Retention Indices |
| --- | --- | --- | --- | --- | --- | --- |
| Diallyl sulfide | 4.103 | 4.000 | 4.313 | 1094127 | 8.78 | 849 |
| Methyl allyl disulfide | 4.967 | 4.853 | 5.110 | 1091824 | 8.76 | a |
| Ethanethioamide,N,N-dimethyl- | 5.699 | 5.557 | 5.773 | 1415789 | 11.36 | 1226 |
| Dimethyl trisulfide | 5.847 | 5.803 | 5.910 | 116087 | 0.93 | 972 |
| 1,4-DIMETHOXYBUTANE | 7.337 | 7.263 | 7.407 | 42649 | 0.34 | a |
| Diallyl disulfide | 7.700 | 7.513 | 7.770 | 3785620 | 30.38 | 1099 |
| Allyl disulfide | 7.903 | 7.813 | 7.950 | 224190 | 1.80 | 1099 |
| Diallyl disulphide | 8.017 | 7.953 | 8.047 | 229038 | 1.84 | 1099 |
| 1,2,3-thiadiazole,5-Methyl- | 8.090 | 8.047 | 8.153 | 115189 | 0.92 | a |
| Methyl allyl trisulfide | 8.910 | 8.760 | 9.007 | 1023967 | 8.22 | a |
| Thiirane, 2-methyl- | 9.300 | 9.193 | 9.427 | 103610 | 0.83 | a |
| 3-Vinyl-3,4-dihydro-1,2-dithiine | 9.937 | 9.830 | 10.007 | 414826 | 3.33 | a |
| Thiazole, 2,4-dimethyl- | 10.233 | 10.110 | 10.270 | 699961 | 5.62 | a |
| 3-Vinyl-1,2-dithiacyclohex-5-ene | 10.347 | 10.273 | 10.393 | 223258 | 1.79 | 1134 |
| Tetrasulfide, dimethyl | 10.433 | 10.400 | 10.470 | 40501 | 0.32 | a |
| Diallyl trisulfide | 11.630 | 11.543 | 11.670 | 559543 | 4.49 | 1350 |
| Propane,2-isothiocyanato-2-methyl- | 11.760 | 11.717 | 11.800 | 56784 | 0.46 | a |
| Diallyl monosulfide | 11.930 | 11.843 | 11.983 | 127320 | 1.02 | 849 |
| dithiolane | 12.603 | 12.497 | 12.653 | 741823 | 5.95 | 882 |
| 1,3-Dithiole-2-thione | 12.777 | 12.700 | 12.827 | 356738 | 2.86 | 1172 |

The chromatographic conditions for the GEO were as follows: chromatographic column: Rtx-5ms column (30 m × 0.25 mmID × 0.25 μm df). Carrier gas: High-purity helium, carrier gas flow rate: 1.0 mL / min, split ratio: 0.0, gasification temperature of 300 ℃. Temperature program: initial temperature: 60 ℃, held for 1.0 min; temperature increased to 120 ℃ at a rate of 10 ℃·min^-1^, held for 2.0 min; temperature increased to 280 ℃ at a rate of 20 ℃ · min^-1^, held for 4.0 min. Injection volume: 1 μL solvent delay time 2.45 min. Ion source: EI source, electron energy: 70 eV, ion source temperature: 250, interface temperature 250 ℃, Scanning mass range: m/z 33-600, mass spectrometry search standard library: NIST05, NIST05s and Wiley7 three libraries.

| CAS | Retention time | Compound | Mol.  weight | RRI |
| --- | --- | --- | --- | --- |
| 592-88-1 | 4.103 | Diallyl sulfide | 114 | 1094127 |
| 2179-58-0 | 4.967 | Methyl allyl disulfide | 120 | 1091824 |
| 631-67-4 | 5.699 | Ethanethioamide,N,N-dimethyl- | 103 | 1415789 |
| 3658-80-8 | 5.847 | Dimethyl trisulfide | 126 | 116087 |
| 13179-96-9 | 7.337 | 1,4-DIMETHOXYBUTANE | 118 | 42649 |
| 2179-57-9 | 7.700 | Diallyl disulfide | 146 | 3785620 |
| 2179-57-9 | 7.903 | Allyl disulfide | 146 | 224190 |
| 2179-57-9 | 8.017 | Diallyl disulphide | 146 | 229038 |
| 50406-54-7 | 8.090 | 1,2,3-thiadiazole,5-Methyl- | 100 | 115189 |
| 34135-85-8 | 8.910 | Methyl allyl trisulfide | 152 | 1023967 |
| 1072-43-1 | 9.300 | Thiirane, 2-methyl- | 74 | 103610 |
| 62488-53-3 | 9.937 | 3-Vinyl-3,4-dihydro-1,2-dithiine | 144 | 414826 |
| 541-58-2 | 10.233 | Thiazole, 2,4-dimethyl- | 113 | 699961 |
| 62488-53-3 | 10.347 | 3-Vinyl-1,2-dithiacyclohex-5-ene | 144 | 223258 |
| 5756-24-1 | 10.433 | Tetrasulfide, dimethyl | 158 | 40501 |
| 2050-87-5 | 11.630 | Diallyl trisulfide | 178 | 559543 |
| 590-42-1 | 11.760 | Propane,2-isothiocyanato-2-methyl- | 115 | 56784 |
| 592-88-1 | 11.930 | Diallyl monosulfide | 114 | 127320 |
| 557-22-2 | 12.603 | dithiolane | 106 | 741823 |
| 930-35-8 | 12.777 | 1,3-Dithiole-2-thione | 134 | 356738 |
